# A67 RISK FACTORS ASSOCIATED WITH CRITICAL CARE AFTER INPATIENT GASTROINTESTINAL ENDOSCOPY: A 5-YEAR TERTIARY HOSPITAL STUDY

**DOI:** 10.1093/jcag/gwac036.067

**Published:** 2023-03-07

**Authors:** M Ghazarian, K K Leung, L W Yu, K Sullivan, A Samman, M Deeb, A Steel, P James

**Affiliations:** 1 Department of Medicine; 2 Division of Gastroenterology and Hepatology; 3 Department of Anesthesiology, University Health Network, University of Toronto, Toronto, Canada

## Abstract

**Background:**

A subset of hospitalized patients will require critical care after their gastrointestinal endoscopy (GIE) and predicting which patients are at high risk of requiring critical care remains an important challenge.

**Purpose:**

To identify protective and aggravating clinical risk factors associated with critical care involvement within 7 days of inpatient GIE in adults and to develop a tool that could assist in risk-stratifying patients at high risk of requiring critical care post-endoscopy.

**Method:**

This was a single-centre retrospective case-control study of adult patients who underwent inpatient GIE while admitted to ward-level care at Toronto General Hospital from years 2015 to 2019. Cases were defined by inpatients who required critical care response team and/or critical care admission within 7 days of GIE, compared to control patients who did not require critical care throughout admission. Chart review and linked secondary sources were used with defined inclusion and exclusion criteria. Both univariate and multivariate analyses were performed comparing patient baseline, clinical history (including cardiovascular, respiratory, other co-morbidities) and endoscopy characteristics.

**Result(s):**

We identified a total of 275 patients with 302 endoscopies as cases and 2069 patient controls who satisfied inclusion criteria. Critical care involvement was most commonly due to cardiovascular-related complications (n=175, 58%) followed by respiratory complications (n=117, 39%). Amongst cases, death occurred in 9 (3%), 25 (9%) and 67 (22%) within 72 hours, 7 days and 30 days respectively post endoscopy. The strongest associations with critical care involvement within 7 days after GIE included a history of discharge from critical care (OR 2.29 CI 1.70-3.04) and/or recent mechanical ventilatory support (OR 2.27 CI 1.30-3.91) in the 30 days prior to endoscopy, having several co-morbidities involving major organ systems (elevated troponin OR 3.20 CI 2.26-4.52, cirrhosis OR 2.5 CI 1.80-3.46, renal dysfunction 2.09 CI 1.57-2.78) and patients admitted under surgical (OR 3.82 CI 2.54-5.71) or transplant services (OR 4.63 CI 2.94-7.26). The majority of adverse events among cases were not found to be complications directly related to GIE (64% unlikely, 20% possible, 9% probable, 7% definite). Patients with a history of pulmonary hypertension (OR 5.68 CI 0.53-60.70) and ASA score III/IV (OR 3.28 CI 1.01-10.73) had the highest odds of probable or definite endoscopy-related adverse events.

**Conclusion(s):**

This study is the largest to date to examine risk factors associated with critical care requirements post GIE in the tertiary care inpatient setting. The risk factors we have identified can be used to create a tool to determine which inpatients may benefit from anesthesia consultation and support during their endoscopic procedure.

**Please acknowledge all funding agencies by checking the applicable boxes below:**

None

**Disclosure of Interest:**

None Declared

